# Increased Risk of Cancer—An Integral Component of the Cardio–Renal–Metabolic Disease Cluster and Its Management

**DOI:** 10.3390/cells14080564

**Published:** 2025-04-09

**Authors:** Eberhard Standl, Oliver Schnell

**Affiliations:** Forschergruppe Diabetes e.V. at Helmholtz Center Munich, Ingolstaedter Landstraße 1, Neuherberg, 85764 Munich, Germany

**Keywords:** cancer, hyperinsulinemia, insulin resistance, oxidative stress, low-grade inflammation, diabetes, cardio–renal–metabolic syndrome

## Abstract

Cancer risk increases by 25 to 250% not only in dysmetabolic obese or overweight people with overt type 2 diabetes but also in individuals with intermediate hyperglycemia (pre-diabetes), with especially pronounced risk of pancreatic or hepatocellular cancer and obesity-related cancers, e.g., colorectal and kidney cancers, bladder cancer in men, and endometrial and breast cancers in women. Cancer may often be present before or upon the diagnosis of diabetes, as there is a common pathogenetic dysmetabolic–inflammatory background with insulin resistance for developing diabetes, cardiorenal disease, and cancer in parallel. The mechanisms involved relate to hyperinsulinemia as a potential carcinogenic priming event with ectopic visceral, hepatic, pancreatic, or renal fat accumulation that subsequently fuel inflammation and lipo-oncogenic signals, causing mitochondrial oxidative stress and deregulation. Moreover, hyperinsulinemia may foster mitogenic MAP kinase-related signaling, which can also occur via IGF1 receptors due to increased free IGF1 levels in obesity. Weight reduction of 10% or more in obese people with diabetes or pre-diabetes, e.g., through intensive lifestyle intervention or bariatric (=metabolic) surgery or through treatment with GLP-1 receptor agonists or metformin, is associated with significantly lower incidence of “diabesity”-associated cancers. In conclusion, there seems to be huge utility in adopting the new “Cardio-Renal-Metabolic-Cancer Syndrome” approach, also looking for cancer at the time of diabetes diagnosis in addition to proactively screening for undiagnosed dysglycemia.

## 1. Introduction

**Figure 1:**
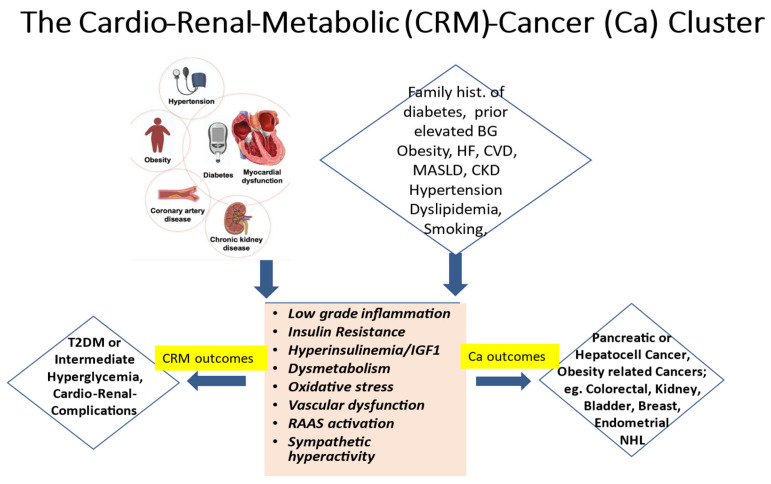
The cardio–renal–metabolic (CMR)–cancer (Ca) cluster. Abbreviations: BG = blood glucose, CKD = chronic kidney disease, CVD = cardiovascular disease, HF = heart failure, IGF1 = insulin-like growth factor 1, MASLD = metabolic dysfunction-associated steatotic liver disease, NHL = non-Hodgkin lymphoma, RAAS = renin–angiotensin–aldosterone system, and T2DM = type 2 diabetes mellitus.

It is widely established that the ongoing global epidemics of obesity and diabetes cause an enormous burden and a still growing challenge for public health in most contemporary societies [1,2]. Driven by excessive body fat accumulation (due to the so-called modern lifestyle with little physical activity but abundant food intake) and against a background of a substantial genetic predisposition present in some 40 to 50% of populations, diabetes mellitus is on the rise worldwide [1,2]. Currently, more than 500 million people are affected. Disturbingly, however, almost half of them are unaware that they are living with the condition. Moreover, some additional 500 million people worldwide exhibit a dysmetabolic pre-stage of diabetes, previously called “prediabetes” and recently renamed as intermediate hyperglycemia. Notably, this stage is characterized by glycemic values above the normal range but below the diagnostic threshold for overt diabetes, yet with a high risk of progressing to the overt disease, besides already showing a multitude of additional adverse cardio–renal–metabolic abnormalities early on, including insulin resistance and low-grade inflammation. The present paper seeks to underpin the notion that increased cancer risk constitutes an integral specific component of this worldwide, abundantly frequent cardio–renal–metabolic disease cluster (see the graphical abstract, Figure 1), with important implications for its appropriate management.

## 2. Epidemiological Notions

In line with the above, epidemiological notions indicate an increased incidence of cancers not only in people with overt diabetes but also in those with intermediate hyperglycemia and related cardio–renal–metabolic (CRM) syndrome [3,4,5,6]. The cancer risk increases by some 25% to 250%, with the risk being especially pronounced for pancreatic or hepatocellular cancers as well as for a wide spectrum of so-called obesity-related cancers, such as colorectal and kidney cancers, bladder cancers in men, and endometrial and breast cancers in women [3,5,6,7,8]. Importantly, though fatal cardiovascular events have declined overall in recent years, people with type 2 diabetes (and to some extent also those with type 1 diabetes) remain at a more than twofold increased mortality risk vs. those without [9], with incident all-cause mortality also relatively increasing because of the increasing number of cancer deaths [9]. Some 50% of all non-cardiovascular deaths in contemporary cohorts with diabetes are attributable to malignancies [10]. Cancer may often be diagnosed even before (i.e., during the stage of “pre-diabetes”) or soon after the diagnosis of diabetes [11], as there is this common pathogenetic dysmetabolic background for developing diabetes, cardiorenal disease, and cancer in parallel [5,12], as already alluded to above (see also Figure 1).

These compelling notions strongly imply a call to clinical action, as they point to a very relevant window of opportunity, particularly in the primary care setting, to proactively consider the possibility of co-existing cancers simultaneously with the diagnosis of diabetes. In addition, and in view of the high number of undiagnosed cases, testing for diabetes and its pre-stages in those at apparent risk (Figure 1) seems likely to offer great merits in attenuating the rolling tsunami of cancer morbidity and mortality [13], apart from being helpful in its own right to prevent serious metabolic decompensation and/or early diabetic complications.

## 3. Diabetes-Specific Pathophysiologic Linkage

It is well established that cancer cells rely on two main energy resources, i.e., oxidative phosphorylation (OXPHOS) and glycolysis [14]. Diabetes mellitus, especially Type-2 diabetes (T2DM), by virtue of the causative mechanisms involved in its pathogenesis, also profoundly impacts OXPHOS and glycolysis due to hyperglycemia-induced hyperinsulinemia in the context of obesity related insulin resistance and dyslipidemia. Thus, the same hallmarks and pathogenetic components within the CRM disease cluster (Figure 1) seem to provide a common ground for developing T2DM, cardiovascular disease (CVD), and a substantial number of “diabesity”-related cancers (Figure 1).

**Figure 2:**
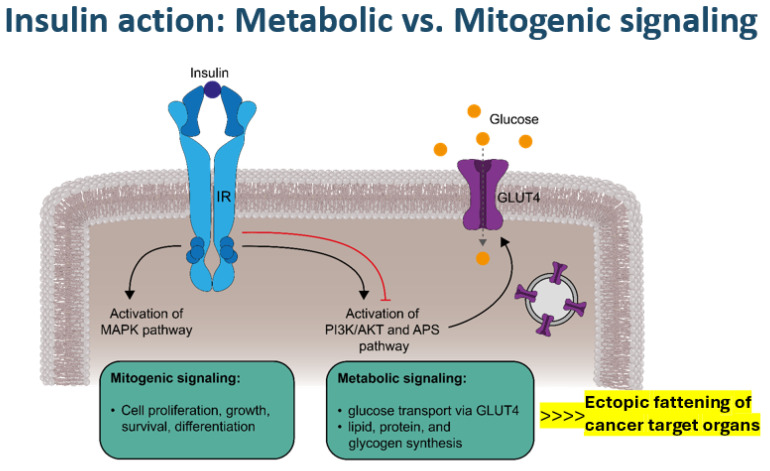
Metabolic versus mitogenic signaling via the insulin receptor (IR). The binding of insulin to its receptor induces two main signaling cascades. Activation of the mitogen-activated protein kinase (MAPK) pathway promotes the mitogenic effects of insulin, such as cell proliferation, growth, survival, and differentiation. Metabolic signaling is mediated by the phosphoinositide-3-kinase (PI3K)/Akt (AKT, also known as protein kinase B) and adapter protein containing a PH and SH2 domain (APS) pathways, which stimulate vesicular glucose transporter type 4 (GLUT4) exocytosis and GLUT4 incorporation into the plasma membrane, enabling cellular glucose uptake, in addition to other metabolic effects, such as triglyceride synthesis, particularly in ectopic adipose tissue. Negative feedback mechanisms (red line) regulate insulin signaling, whereas in the context of insulin resistance, mitogenic signals are not interrupted.

Notwithstanding the complex and multifaceted genetic or environmental risk factors involved in cancer development in general, the laid out specific diabetes-related pathophysiologic foundation, on the one hand, may set the scene for a potential carcinogenic priming effect with excessive ectopic visceral, hepatic, pancreatic, or renal fat accumulation [8,12,14,15,16] (Figure 2) that subsequently fuel lipo-oncogenic signals, causing mitochondrial oxidative stress and deregulation. On the other hand, hyperinsulinemia may foster the potential of (mitogenic) mitogen-activated protein (MAP) kinase-related signaling (Figure 2), which can also occur via IGF1 receptors due to increased free insulin-like growth factor-1 (IGF1) levels in obesity [17,18]. Oncogenic product formation in the tricarboxylic acid (TCA) cycle may evolve due to mitochondrial oxidative stress and gene deregulation [19,20] in the presence of abundant fatty acid availability that leads to a shift to fatty acid usage as a less effective fuel for energy production compared with glucose [14,21]. The excessive fatty acid utilization may yield (DNA-)toxic free radical formation that can affect the redox control of cell growth and death [22,23], as well as cause mitochondrial dysfunction, especially dysfunctionality of the carnitine shuttle in the mitochondrial membrane [14,19,20,21,22,23,24], as reflected by specific metabolite signatures [21,24]. Both “reprogramming of energy metabolism” and disordered mitochondrial outer membrane permeabilization have been shown to represent key features of potential malignant cell transformation [14,17,19,20,21,22,23,24]. Relative hyperinsulinemia is most pronounced prior to the manifestation of overt type 2 diabetes and in obese individuals, consistent with the epidemiological observations regarding the timing of diabetes-related cancer manifestations discussed above.

Activation of MAP kinases not only by mitogenic insulin signaling but also by IGF1 signaling [18], together with overexpression of epithelial growth factor (EGF) or vascular endothelial growth factor (VEGF) in adipose tissue in obesity, may form a strong cell proliferation and growth stimulation scenario [17,18,19,21,24,25]. Oxidative stress with an altered intracellular redox state, endoplasmic reticulum stress, and hyperglycemia-related NFK-ß formation may occur alongside the inhibition of AMP kinase and the reduced expression of tumor-suppressor genes [21,24,25,26,27]. Notably, a sedentary lifestyle not only represents an important pro-oxidant factor but also a strong obesogenic and diabetogenic factor [1,2,8]. Looking at dietary factors, a prospective study performing diet-wide analyses of the risk of colorectal cancer in 12,251 incident cases among 542,778 women in the UK observed a strong positive association with red and processed meat intake and alcohol consumption, whereas the intake of dairy containing calcium combined with the ingestion of cereal, fruit, wholegrains, fiber, folate, and vitamin C showed protective effects [28]. Against this background, excess fat content inside or surrounding organs plays a crucial amplifying role in rendering the involved organs a particular target of potential cancer transformation (Figure 2). These specific “Diabesity”-related tumorigenic mechanisms converging to oncogenic mitochondrial gene deregulation seem to apply in particular to the development of pancreatic, hepatocellular, renal, colorectal, breast, and bladder cancer and perhaps also non-Hodgkin lymphoma [12,15,17,21,25,26,27,29]. Of note, IGF1 signaling seems to be especially involved in colorectal and breast cancer [18], while hyperglycemia-associated dysregulation of the endothelin system has been recognized to play an important role in the biology of tumors such as those of the bladder, breast, and colon [30].

The reprogramming of mitochondrial energy metabolism is a well-known hallmark of cancer formation [19,20]. Mitochondrial dysfunction can promote malignant transformation, i.e., the conversion of a healthy cell into a malignant precursor, as a consequence of (1) reactive oxygen species (ROS) overgeneration, which favors mutagenesis; (2) accumulation of succinate, fumarate or 2-hydroxyglutarate generated in the TCA cycle, all of which can operate as oncometabolites; and/or (3) increased resistance to oncogene-driven mitochondrial outer membrane permeabilization (MOMP)- or mitochondrial permeability transition (MPT)-driven regulated cell death or cellular senescence [19,20,31]. All of this can be triggered by diabetic dysmetabolism, as it specifically leads to a considerable mitochondrial fuel overload by shifting to predominant fatty acid oxidation, i.e., a shift to altered substrate metabolism, causing lipo- and gluco-toxicity, ROS production, and oxidative stress [8,27,31]. These cancer pre-dispositioning mechanisms are likely in excessive operation during the state of pre-diabetes, during which hyperinsulinemia and augmented triglycerides synthesis are most prominent, in line with the epidemiological notions presented above.

**Figure 3:**
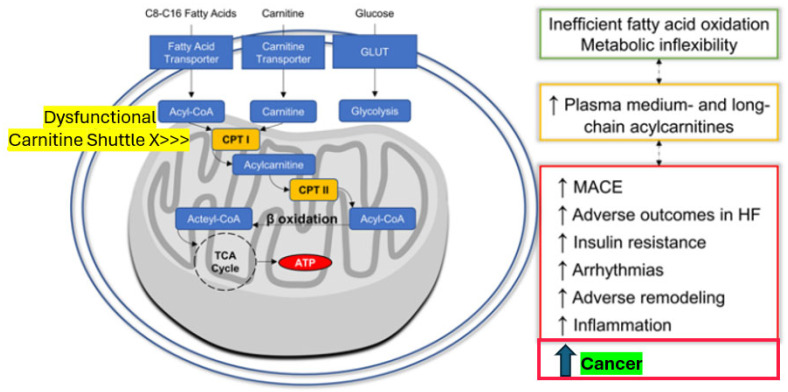
The novel cardio–renal–metabolic–cancer disease-related paradigm: Mitochondrial dysfunction induced by excessive yet dysregulated fatty acid oxidation is associated not only with MACE, HF, and arrhythmia events but also with cancer (modified from 30). Abbreviations: Acyl-CoA = acyl-coenzyme A, ATP = adenosine-tri-phosphate, CPT I (or II) = carnitine palmitoyl-transferase I (or II), HF = heart failure, MACE = major adverse cardiovascular events, and TCA Cycle = tricarboxylic acid cycle (citric acid).

Of further note, similar convergent mechanisms also seem to be involved in the pathogenesis of the cardiovascular complications of diabetes. We have recently found compelling evidence that mitochondrial metabolites indicating dysregulated fatty acid metabolism predict cardiovascular events in individuals with type 2 diabetes [32]. Medium- and long-chain acyl-carnitines showed a distinct metabolomic signature in study participants who later developed major adverse cardiovascular events, including heart failure. These abnormalities at the individual medium-chain acyl-carnitine level most probably reflect disordered mitochondrial transmembrane fatty acid transport via the carnitine shuttle, thus creating inefficient fatty acid oxidation and metabolic inflexibility connected with adverse tissue remodeling, insulin resistance, inflammation, and cardiovascular disease (Figure 3). Leptin signaling with the generation of cytokines such as interleukin 1 (IL1), IL6, tumor necrosis factor@ (TNF@), visfatin, resistin, and monocyte chemoattractant protein-1 (MCP-1) and priming of immune cells plays a pivotal role in this inflammatory response [33,34]. In addition, adverse signals coming from the gut, like toxins contained in food that yield free radicals or mediated via the microbiome, fostering inflammation, are of importance [33,35,36]. Triggered by chronic hyperglycemia and driven by subsequent increased formation of advanced glycation end products as well as ROS, the nucleotide-binding domain, leucine-rich-containing family, pyrin domain-containing-3 (NLRP3) inflammasome has been uncovered as a key player not only in the development of diabetes but also its complications, including cancer [33].

Interestingly, randomized diabetes treatment with exenatide, which has both anti-inflammatory and fat mass-reducing properties among its complex pleiotropic action portfolio, was associated with a reversal of the observed metabolomic abnormalities in our study [32]. Therefore, on one side, adverse fatty acid metabolism-derived signals, especially from ectopic perivascular or epicardial fat, seem to further cardiovascular disease. On the other side, they might induce cancer cell transformation (see also Figure 3) if released peri-viscerally to the colon or from ectopic fat tissue inside the pancreas, the liver, the kidneys, or the female breast, inducing target organ inflammation [21,24,25,26,27] connected with mitochondrial dysfunction. With this unifying concept in mind, effectively reducing excessive and inflamed adipose tissue should be a promising opportunity to also minimize the intrinsic cancer risk [18,20,26,32].

## 4. Preventive Strategies and Diabetes Therapy-Related Aspects

Indeed, reducing critical body fat mass along with reverting to normoglycemia and eumetabolism has provided compelling evidence that a reduction in body weight of 10% or more in obese people with diabetes or pre-diabetes, e.g., through bariatric (=metabolic) surgery [37,38] or through the use of GLP-1 RA-based therapies (best in combination with metformin 21,24, see below), is associated with lower risks of specific types of obesity-associated cancers [39,40]. The “proof-of-principle” demonstrating bariatric interventions showed a relative risk reduction of about 50% for all cancers, including hepatocellular and pancreatic cancers with some 65% relative risk reduction [37,38]. The accompanying sustained weight loss amounted to approximately 25 kg. More or less a similar margin of decreased cancer risk was seen with GLP1-RAs or metformin, though the attained weight loss was less impressive [21,24,39]. Interestingly, it has been suggested that GLP-1 RAs, by means of calorie restriction, exert immediate and multifaceted effects by activating AMP kinase, thus, e.g., stimulating the expression of tumor-suppressor-genes [40].

Recently, the DiRECT study also published very encouraging results at its five-year follow-up examination regarding the beneficial effects of sustainable weight loss in diabetes [41]. In DiRECT, a randomized controlled effectiveness trial, an intensive weight management intervention resulted in a mean weight loss of 7.6 kg after 2 years, with 36% of participants in remission of type 2 diabetes. Of the 36 participants in the intervention group who maintained over 10 kg weight loss at 2 years, 29 (81%) were in remission. Continued low-intensity dietary support was then offered up to 5 years from baseline to intervention participants, aiming to maintain weight loss and gain clinical benefits. Regarding cancer, eight newly diagnosed cancers (two colon, two prostate, and one each of pancreas, lung, kidney, and bladder) developed in the non-extension control group, whereas only one (lung) occurred in the extension intervention group in a participant who had withdrawn before their diagnosis. These data, though observational, strongly suggest that reaching remission of diabetes is an important new management target, achievable by many people living with type 2 diabetes, and even preventive for cancer, on top of reducing cardiovascular complications [41]. At the brink of the second quarter of the 21st century, more and more effective tools seem to be available to achieve this goal [42]. Overall preventive strategies and diabetes therapy-related aspects are summarized in Table 1.

Beyond sufficient weight loss, following specific dietary rules seems helpful, as already alluded to above. Avoiding red and processed meat intake and restricting alcohol intake per day to below 30 g for men and below 20 g for women show proven benefits [12,28,43]. Conversely, ingestion of fruit, wholegrains, fiber, vegetables, diet-contained folate, riboflavin, and vitamin C (the latter two recycle oxidized glutathione) and dairy containing calcium at more than 1000 mg/d might be recommended [28]. In this context, intake of high-dose synthetic folate should be cautioned, however, as, on the contrary, excessive concentrations may elicit tumor progression. The presence of natural antioxidants in the diet, however, has been directly related to a lower incidence of cancer [27]. Whether supplementation with probiotics confers additional advantages is a matter of ongoing debate, though they can effectively lower inflammatory factors such as IL-1 ß or IL6, marginally improve glycemic control, and alleviate metabolic syndrome, but the effects are small [44,45,46].

The requirements of appropriate therapy with glucose-lowering drugs in the context of cancer and diabetes have recently been reviewed in several publications [8,47]. Without being repetitious, yet exquisitely focusing on the conceptual approach proposed in this paper, the ideal drug should be able to more or less “cure” the adverse common ground of the obesity-related CRM–cancer disease cluster (in combination with appropriate lifestyle management, including dietary recommendations, as mentioned above). In terms of drug characteristics, this means ensuring sustainable weight loss and effective removal of ectopic dysfunctional adipose tissue on the one side and near normalization of blood glucose on the other side. If possible, glycemic control strategies should aim for the remission of diabetes, yet without stimulating hyperinsulinemia, but rather due sensitization of insulin target tissues. Moreover, normalizing the intracellular redox state by manipulating OXPHOS genes and enhancing mitochondrial complex I activity or by adjusting the metabolite production in mitochondria, respectively, would be welcome to restrain tumor growth [14,23]. For example, mechanisms engaged in increasing NAD+/NADH levels effectively prevent metastasis and the progression of cancer [14,23]. In essence, metformin, GLP-1 RAs, and SGLT2 inhibitors might be good candidates to fulfill these prerequisites and could be nearly as effective as bariatric (=metabolic) surgery in reducing cancer morbidity and mortality [37,38].

To compare the incident risk of each of the 13 obesity-associated cancers (OACs) in patients with T2D who were prescribed GLP-1 RAs vs. insulin or metformin, a recent cohort study in the US evaluated some 1.6 million patients with T2D who had no prior diagnosis of the 13 OACs [39]. The results indicated that patients with T2D treated with GLP-1 RAs vs. insulin had a significant risk reduction (by about 25 to 65%) in 10 of the 13 OACs, including esophageal, colorectal, endometrial, gallbladder, kidney, liver, ovarian, and pancreatic cancer. No difference in cancer risk was associated with GLP-1 RAs compared with metformin, suggesting similar efficacy of GLP-RAs and metformin in reducing cancer risk compared with patients on insulin therapy. Bias by indication, of course, can never be excluded despite propensity score matching of baseline covariates, but the numbers are impressive and support the case both of metformin and/or GLP1-RA use for glycemic control in people with CRM and potential cancer issues. Also of note, no negative signal regarding thyroid cancer was observed in connection with GLP1-RA use in this huge cohort database. Mechanistically, it has been shown that treatment with GLP-1 RA reduced ROS production and recovered mitochondrial membrane potential, mitochondrial respiration, and myeloperoxidase (MPO) levels in persons with type 2 diabetes [48], in line with our earlier findings regarding mitochondrial metabolomics [32].

Expanding on metformin, a reduced cancer risk with metformin has been described in a number of observational studies and in conjunction with reduced plasma insulin levels [21,24,39,49,50,51]. Transcriptomic analysis of human primary breast cancer has identified fatty acid oxidation as a target for metformin [21], relating to two adaptation pathways to the drug [24] that attenuate oncogenic signals to the mitochondria discussed above. Interestingly, metformin has been found to increase OXPHOS-relevant gene transcription and mitigate NLRP3 inflammasome activity [24].

In terms of sodium–glucose transporter-2 (SGLT2) inhibitors, findings collectively indicate that there is overexpression of SGLT2 transporters in most tumors, presenting a potential therapeutic target for specific inhibitors against this protein [52]. In addition, SGLT2 inhibitors block the Wnt/β-Catenin pathway, which plays a pivotal role in the development of liver cancer. Conversely, like GLP1-RAs [40], SGLT2 inhibitors activate the AMP-dependent protein kinase (AMPK) pathway, which comprises a crucial signaling mechanism regulating cellular growth, e.g., by suppressing fatty acid synthesis as well as proliferation and survival among cancer cells [52]. Looking at clinical outcomes, a recent meta-analysis has shown that in cancer patients, the use of SGLT2 inhibitors can also significantly reduce the risks of all-cause mortality, heart failure hospitalization, or clinically significant arrhythmias, demonstrating their established CV benefit in these patients as well [53]. A systematic review and meta-analysis of randomized, double-blind, placebo-controlled trials with SGLT2 inhibitors, however, revealed a 39% higher risk of renal cancers with SGLT2 inhibitors compared with a placebo [54], though the overall cancer risk was not increased in the SGLT2 inhibitor group [54]. The evaluation of incident cancer risk, notably bladder cancer and breast cancer, in patients with heart failure with and without diabetes treated with SGLT2 inhibitors [55] rated the results as inconsistent and requiring further big data analyses [55]. In all, given the life-saving and mortality-reducing benefit of SGLT2 inhibitors in patients with heart failure, chronic kidney disease, and CVD [56], which currently is unequivocally acknowledged in all international guidelines, watchful monitoring of the millions of patients on SGLT2 inhibitor therapy should be undertaken, keeping the common ground concept of the high-risk CRM–cancer cluster in mind.

Looking further at diabetes medications where there have been some concerns in the past, it seems fair to state that contrary to earlier fears, there seems to be no increased risk related to the use of insulin glargine, as evidenced in randomized controlled studies [57,58], or other forms of insulin, whilst bias by indication may play a role in observational data [39,58]. Likewise, no increased cancer risk due to sulfonylureas was apparent in a recent cohort study [59], but in light of the discussed contributory effect of hyperinsulinemia to the CRM–cancer connection, neither sulfonylureas nor insulin represent a prioritized treatment option for glucose control in patients at risk of cancer. Moreover, in frail patients with type 2 diabetes on insulin (but not in those on sulfonylureas), a relationship between cancer and severe hypoglycemic events has been shown, alluding to a specific multimorbid patient phenotype that needs to be recognized, as it poses challenging management issues, including de-intensification of glucose-lowering therapies [60,61,62].

Regarding Dipeptidyl Peptidase-4 (DPP-4) inhibitors, by virtue of its possible action on many proteins, a wealth of mechanisms have been discussed with potential influence on cancer biology, both as a suppressor as well as an inducer of tumor transformation and proliferation [63]. A meta-analysis of 72 randomized controlled clinical trials, however, encompassing 70,000 enrolled participants, did not substantiate significant associations between the use of DPP-4 inhibitors and cancer development in comparison with the use of other active drugs or placebo [64]. The results were consistent across pre-defined subgroups stratified by type of DPP-4 inhibitor, type of cancer (including pancreatic and thyroid cancer), drug for comparison, trial duration, or baseline characteristics [64]. Therefore, also in this context, further watchful observation seems reasonable, though their insulinotropic action puts DPP-4 inhibitors not high on the list of preferred diabetes medications in patients at risk of cancer.

## 5. Conclusions and Perspectives

In all, there seems to be huge utility in adopting the new concept of a “Cardio-Renal-Metabolic-Cancer Syndrome” approach in the primary care setting where most of these one billion people potentially at risk are seen; also looking for cancer in individuals with other hallmarks of the syndrome is strongly warranted, especially upon the diagnosis of diabetes. Cancer is emerging as the next management frontier after tackling cardiovascular diseases in people with or at risk of diabetes mellitus. Appropriate cancer screening at the population level is highly (cost) effective in view of a recent US analysis in people over 45 years, with several million more deaths averted in this group than by treatment alone [65]. The new concept may also have far-reaching consequences at the level of cardiology specialists, as, e.g., a recent nationwide study in Denmark clearly showed a rapidly increasing temporal trend in the incidence of malignancy in heart failure patients, regardless of co-existing diabetes [66]. To this end, following the integrated “Cardio-Renal-Metabolic-Cancer Syndrome” thinking in the clinical care of those and other cardiological patients should be fundamental so as not to jeopardize the tremendous gains in disease survival time in recent years due to modern guideline-based therapies [56].

## Figures and Tables

**Table 1:** Addressing increased cancer risk connected with dysglycemia: Preventive strategies and diabetes therapy-related aspects.

Consider the impact of the increased risk of co-existing cancer on the diagnosis of any degree of dysglycemia.
Consider screening for undiagnosed dysglycemia in people at high risk of cardio–renal–metabolic (CRM) diseases, particularly in people with a family history of diabetes, prior elevated blood glucose readings, obesity, heart failure, CVD, MASLD, CKD, hypertension, dyslipidemia, or smoking.
Aim for early reversion of dysglycemia through appropriate lifestyle management and reducing body weight by 10% or more in obese or overweight people.
Avoid red and processed meat intake and restrict alcohol intake per day to below 30 g for men and below 20 g for women.
Prioritize the ingestion of fruit, wholegrains, fiber, vegetables, diet-contained folate, riboflavin, vitamin C, and dairy containing calcium.
Consider bariatric (=metabolic) surgery in obese dysglycemic people refractory to conservative therapy.
Prioritize the early use of GLP1 receptor agonists and metformin for weight loss and glycemic management.
Additional combined use of SGLT2 inhibitors may be highly warranted because of their outstanding CRM benefits, yet it is necessary to keep in mind some potential uncertainties regarding urinary tract cancers.
DPP4 inhibitors may be useful additional oral glucose-lowering medications.

## Data Availability

Not applicable.
